# Supplementary material for: Estuarine plastisphere as an overlooked source of N2O production
Source: Nat Commun. 2022 Jul 6;13:3884. doi: 10.1038/s41467-022-31584-x (PMC9259610; doi:10.1038/s41467-022-31584-x)
Supplement: Supplementary file 1 — Supplementary Information [file 41467_2022_31584_MOESM1_ESM.pdf]

SUPPLEMENTARY INFORMATION to

Estuarine plastisphere as an overlooked source of N_2O production

Xiaoxuan Su[1,2,※], Leyang Yang[1,3,※], Kai Yang[1], Yijia Tang[4], Teng Wen[5], Yingmu Wang[6], Matthias C. Rillig[7,8], Lena Rohe[9], Junliang Pan[10], Hu Li[1], Yong-guan Zhu[1,3,11] *

[1] Key Laboratory of Urban Environment and Health, Institute of Urban Environment, Chinese Academy of Sciences, 1799 Jimei Road, Xiamen 361021, China

[2] Interdisciplinary Research Center for Agriculture Green Development in Yangtze River Basin, College of Resources and Environment, Southwest University, Chongqing, 400715, China

[3] University of the Chinese Academy of Sciences, 19A Yuquan Road, Beijing 100049, China

[4] School of Life and Environmental Sciences, The University of Sydney, NSW 2015, Australia

[5] School of Geography, Nanjing Normal University, Nanjing 210023, China

[6] College of Civil Engineering, Fuzhou University, Fuzhou 350116, China

[7] Freie Universität Berlin, Institute of Biology, Berlin, Germany

[8] Berlin-Brandenburg Institute of Advanced Biodiversity Research, Berlin, Germany

[9] Thünen Institute of Climate-Smart Agriculture, Bundesallee 65, Germany

[10] School of Electrical Engineering, Chongqing University, Chongqing 400044, China.

[11] State Key Laboratory of Urban and Regional Ecology, Research Center for Eco-Environmental Sciences, Chinese Academy of Sciences, Beijing 100085, China

※ These authors contributed equally: Xiaoxuan Su, and Leyang Yang.

* Corresponding author: Yong-guan Zhu (ygzhu@rcees.ac.cn); Phone: 86-592-6190997; fax: +86 592-6190977; Address: 1799 Jimei Road, Xiamen 361021, China.

Supplementary Methods

1 Water characteristics

Ammonium, nitrate and nitrite concentrations in the bulk water were detected by an Ion Chromatograph (ICS-3000, Dionex, USA) after being filtered with a 0.22μm membrane. Iron concentrations were measured by an ICP-MS (7500cx, Agilent, USA) . Total organic carbon (TOC) concentrations were detected by a TOC Analyzer (Elementar, Germany).

2 Scanning electron microscope (SEM)

The obtained plastic fragments (3mm×3mm) were washed twice with 4°C 0.05 M phosphate buffer for 10min, and then fixed in 1 mL phosphate buffer containing 2.5% glutaraldehyde at 4°C for 12 h. The mixture was washed twice with the phosphate buffer to remove glutaraldehyde, and then was dehydrated through cold ethanol serials (30%, 50%, 70%, 90%, and 100%, 10 min/step). The plastic fragments were subsequently transferred to a tin foil and critical-point dried (Autosamdri 815 automatic critical-point dryer; Tousimis, USA) for 12 h. The dried plastics were adhered to the conductive adhesives (SPI, USA), sputter coated with gold, and then imaged by SEM (Hitachi S4800, Japan) at 20 kV.

Supplementary Table 1. Information of co-occurrence network analysis for three types of denitrifiers in the Plastisphere and bulk water.

Characteristics	Plastisphere	Bulk water
Numbers of nodes	65	58
Numbers of edges	596	310
Positive (%)	52.78	66.23
Negative (%)	47.22	33.77
Cluster coefficient	0.543	0.612
Graph density	0.219	0.148
Mean degree	14.478	10.010
Mean path length	1.972	2.209
Modularity	0.87	0.69

Supplementary Table 2. Mean values and errors of estimated by Monte Carlo sampling method N_2O-SP (site preference) and N_2O-$\delta^{18}O$, which were used for the N_2O isotope mass balance.

Endmembers	SP (‰)	$\delta^{18}O$ (N_2O/H_2O) (‰)	Supplementary References
BD	-1.5 ± 3.9	19 ± 3.6	[1-3]
FD	37 ± 3.2	47 ± 6.2	[2, 4]
CD	16 ± 4.1	30 ± 6.3	[5-7]
N_2O reduction	ε_{SP}: -6	ε_{18O}: -25	[2, 8]

Note: BD is **b**acterial **d**enitrification; FD is **f**ungal **d**enitrification; CD is **c**hemo**d**enitrification.

Codes for Gaussian estimation and Monte Carlo sampling

```
%------Calculations of Mean values and errors of N2O-SP and N2O-δ18O for three endmembers-------
Amean=mean(abcefg(:,1)); %A: SPBD
Astd=std(abcefg(:,1));
Bmean=mean(abcefg(:,2)); %B: SPFD
Bstd=std(abcefg(:,2));
Cmean=mean(abcefg(:,3)); %C: SPcD
Cstd=std(abcefg(:,3));
Emean=mean(abcefg(:,4)); %E:  δ18OBD
Estd=std(abcefg(:,4));
```

```
Fmean=mean(abcefg(:,5)); %F:  δ18OFD

Fstd=std(abcefg(:,5));

Gmean=mean(abcefg(:,6)); %G:  δ18OCD

Gstd=std(abcefg(:,6));


%------Main Program-------

num_sample=40000; %Total stimulations

num_sample1=10000;%effective stimulations

result=zeros(15,4);

for nnn=1:15

num_data=nnn;

j=1;

f123=ones(num_sample1,3);

%------Random sampling of SP and  δ18O -------

A=normrnd(Amean,Astd,[1 num_sample]);

B=normrnd(Bmean,Bstd,[1 num_sample]);

C=normrnd(Cmean,Cstd,[1 num_sample]);

E=normrnd(Emean,Estd,[1 num_sample]);

F=normrnd(Fmean,Fstd,[1 num_sample]);

G=normrnd(Gmean,Gstd,[1 num_sample]);


%------Calculations of fBD, fFD, and fCD------

for i=1:num_sample

H=[1 A(i) E(i); 1 B(i) F(i);1 C(i) G(i)];

if max(data(num_data,:)*inv(H))<1 && min(data(num_data,:)*inv(H))>-1

f123(j,:)=data(num_data,:)*inv(H);
```

```
j=j+1;
end
if j>num_sample1
    break
end
end
%------Results output-------
f123mean=mean(f123);
result(nnn,1:3)=mean(f123);
result(nnn,4)=sum(sum((f123-f123mean).^2))/(num_sample1-1);
end

%------Figuring-------
% hold on
% for i=1:10000
%       if max(f123(i,:))<1 && min(f123(i,:))>-1
% %           if   min(f123(i,:))<0
% %           plot(f123(i,1),f123(i,2),'m*')
% %           else
% %           plot(f123(i,1),f123(i,2),'r*')
% %           end
%       plot(f123(i,1),f123(i,2),'r.')
%       end
% end
% plot(f123mean(1),f123mean(2),'b*')
```

Supplementary Table 3. Primers for targeting the bacterial and fungal communities in the plastisphere and bulk water.

Genes	Primers	Supplementary References
b*nirK*	F1aCu ATCATGGTSCTGCCGCG<br>R3Cu GCCTCGATCAGRTTGTGGTT	[9]
b*nirS*	Cd3aF GTSAACGTSAAGGARACSGG<br>R3cd GASTTCGGRTGSGTCTTGA	[9]
ITS	CTTGGTCATTTAGAGGAAGTAA<br>GCTGCGTTCTTCATCGATGC	[10]

Supplementary Table 4. Primers of qPCR-based Smartchip analysis for bacterial denitrifying functional genes in the plastisphere and bulk water.

Genes	Primers	Supplementary References
narG	TAYGTSGGGCAGGARAAACTG<br>CGTAGAAGAAGCTGGTGCTGT	[11]
nirS	GTSAACGTSAAGGARACSGG<br>GASTTCGGRTGSGTCTTGA	[9]
nirK	ATCATGGTSCTGCCGCG<br>GCCTCGATCAGRTTGTGGTT	[9]
nosZ	CGCRACGGCAASAAGGTSMSSGT<br>CAKRTGCAKSGCRTGGCAGAA	[12]

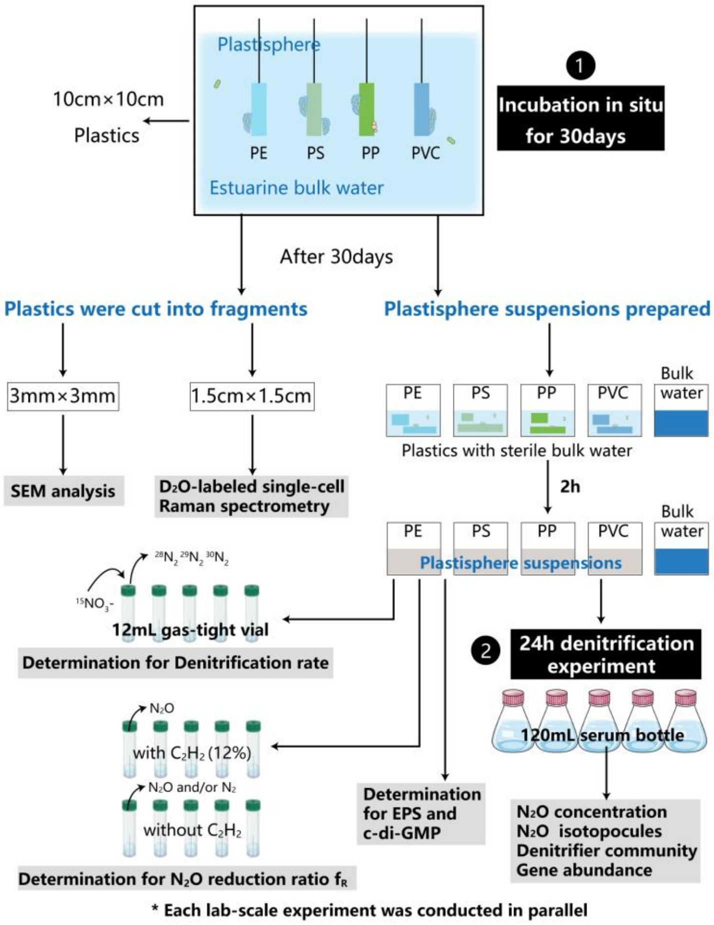

Supplementary Fig. 1. Experimental design. We selected four types of plastics. This study included (1) *in situ* incubation for 30d, and (2) denitrification experiment for 24h. During the 30d *in situ* incubation, the 10×10cm plastics debris was connected by cotton cords and suspended at 4m below the estuarine water. After 30d, a portion of plastics and bulk water was used for water quality analysis, SEM, and heavy water (D_2O)-labeled single-cell Raman spectroscopy. The prepared plastisphere suspensions were used for the synchronous detections of denitrification rate, N_2O reduction ratio, extracellular polymeric substances (EPS) and bis(3′-5′)-cyclic dimeric guanosine monophosphate) (c-di-GMP) levels. During the 24h denitrification experiment, we established plastisphere groups and bulk water group. After 24h, N_2O concentration and isotopocules, denitrifier community, and denitrifying gene abundance in the plastisphere and bulk water were measured.

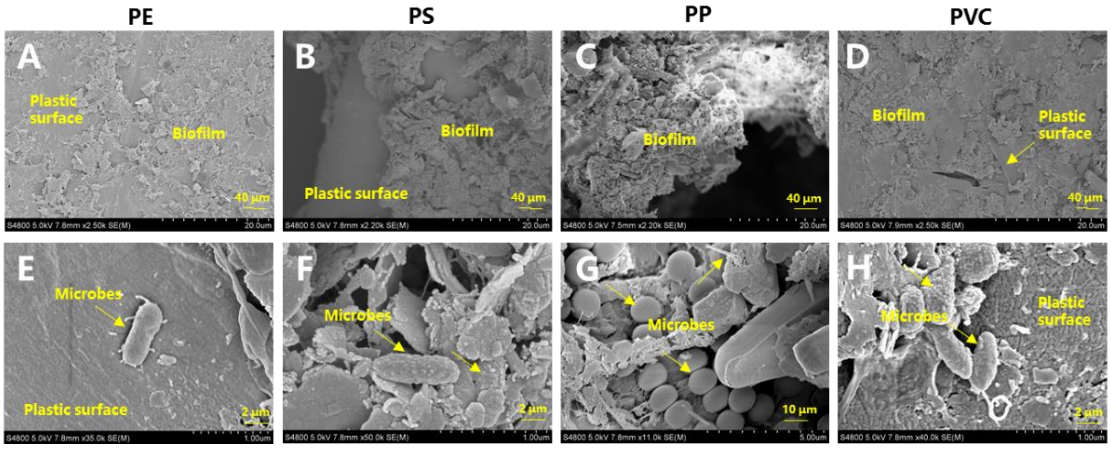

Supplementary Fig. 2. SEM images of biofilm structures on the four types of plastic debris. A-D. Biofilms on the plastics. **E-H.** Diverse microbes observed in the biofilms.

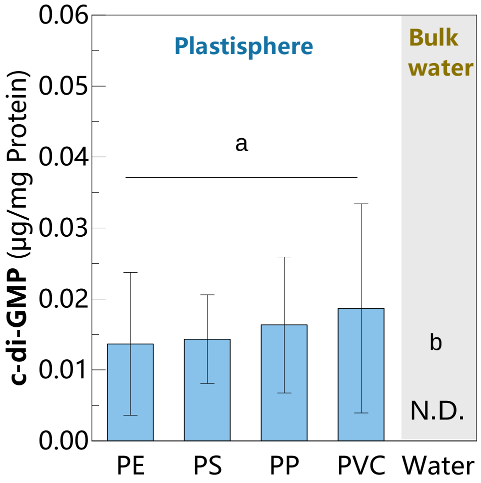

Supplementary Fig. 3. Concentrations of intracellular c-di-GMP in the plastisphere and bulk water. N.D. is non-detectable. Error bars are the standard error. Different letters (a and b) indicate the significant differences ($P<0.05$) among each plastisphere group and bulk water group.

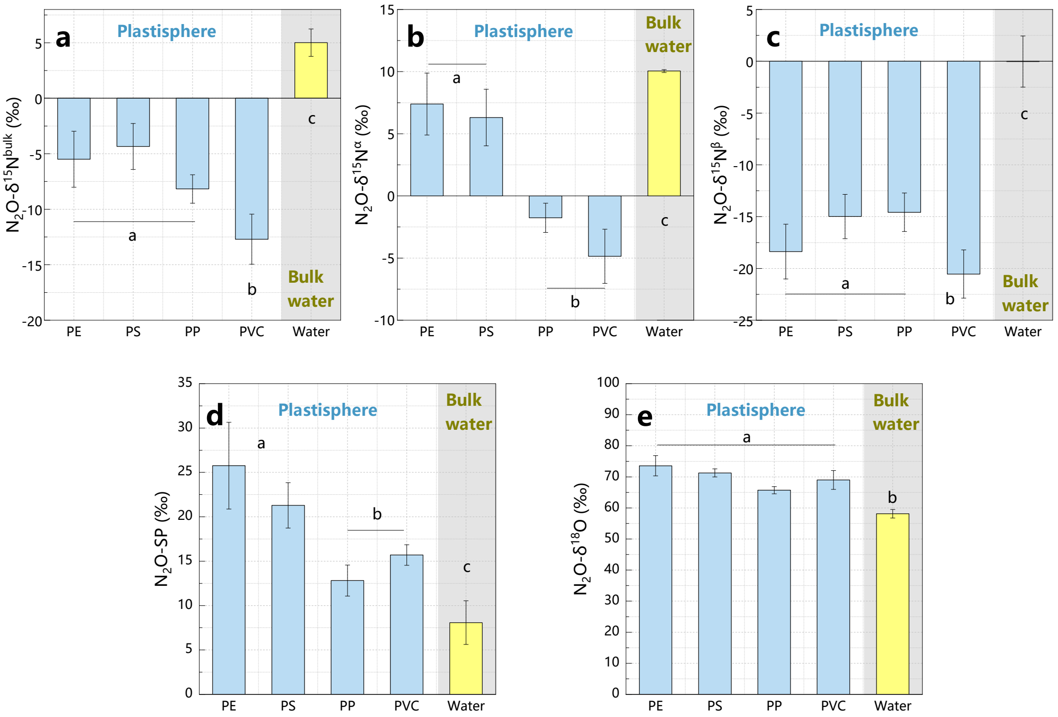

Supplementary Fig. 4. Isotopic compositions and abundances of N_2O produced in the plastisphere and bulk water. (a). $N_2O-\delta^{15}N^{bulk}$. **(b).** $N_2O-\delta^{15}N^{\alpha}$. **(c).** $N_2O-\delta^{15}N^{\beta}$ **(d).** N_2O-SP. **(e).** $N_2O-\delta^{18}O$. Error bars are the standard error. Different letters (a, b, c, and d) indicate the significant differences ($P<0.05$) between Plastisphere and bulk water.

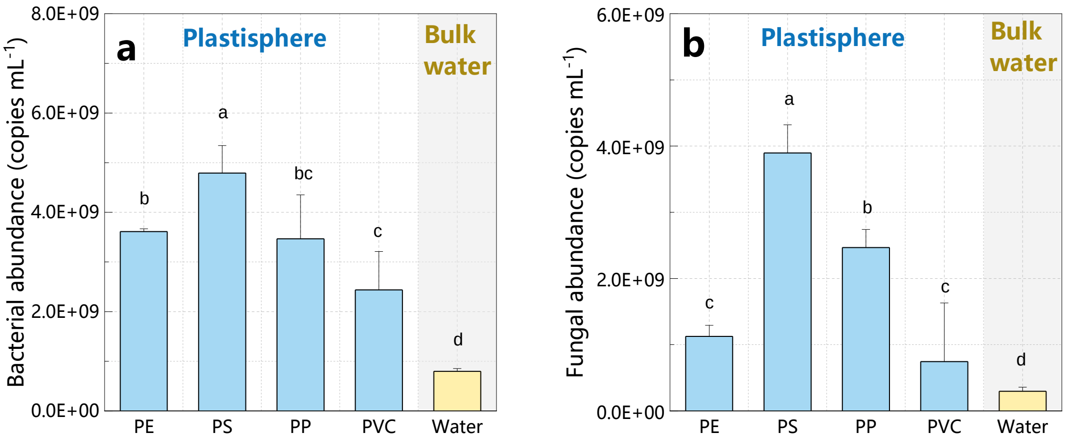

Supplementary Fig. 5. The absolute abundances of 16S rRNA (a) and ITS (b) genes in the plastisphere and bulk water. Error bars are the standard error. Different letters (a, b, c, and d) indicate the significant differences ($P<0.05$) among each plastisphere group and bulk water group.

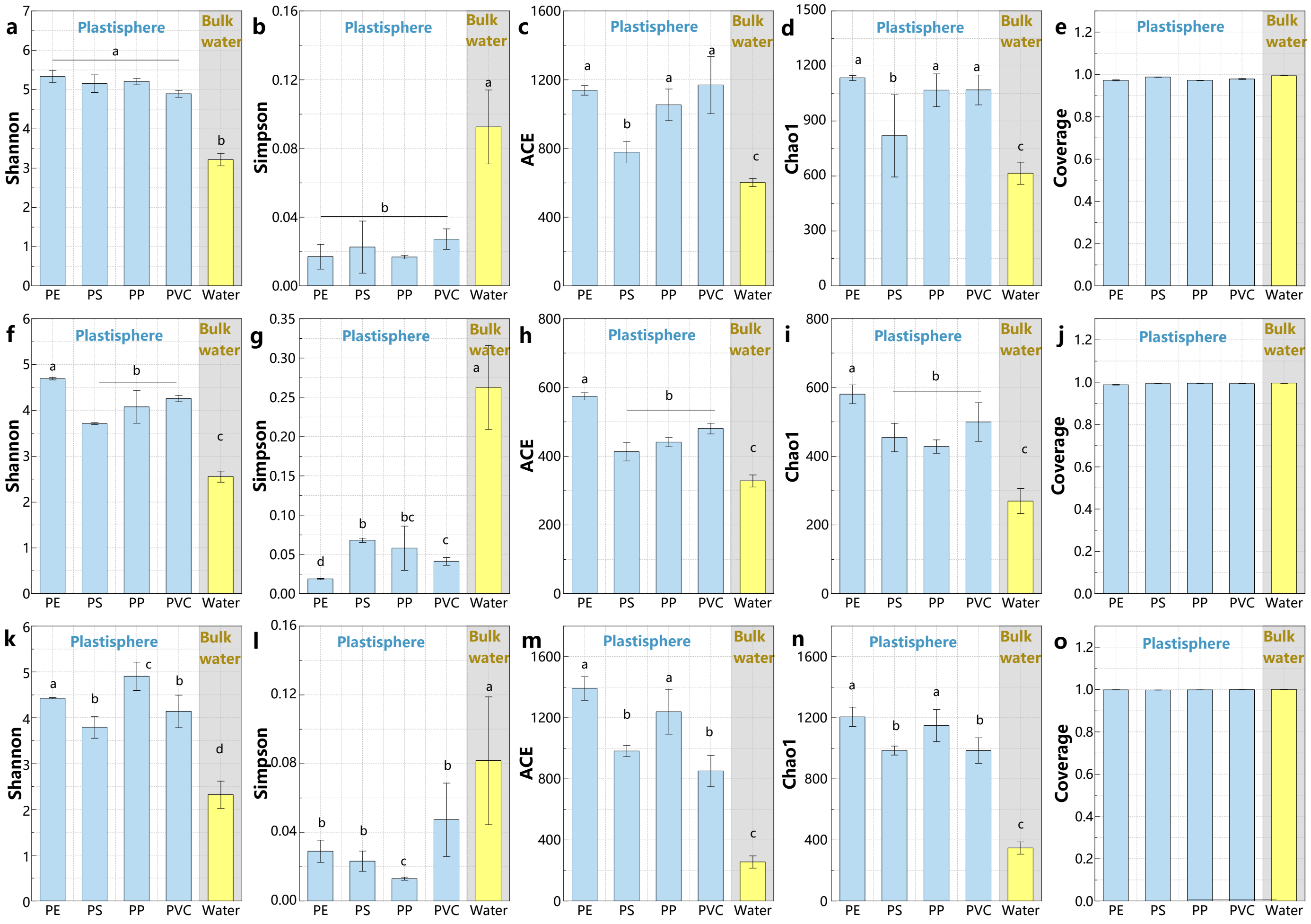

Supplementary Fig. 6. Alpha diversity of the three denitrifier communities in the plastisphere and bulk water. **(a-e).** b*nirS*-type denitrifiers. **(f-j).** b*nirK*-type denitrifiers. **(k-o).** f*nirK*-type denitrifiers. Error bars are the standard error. Different letters (a, b, c, and d) indicate the significant differences (P<0.05) between plastisphere and bulk water.

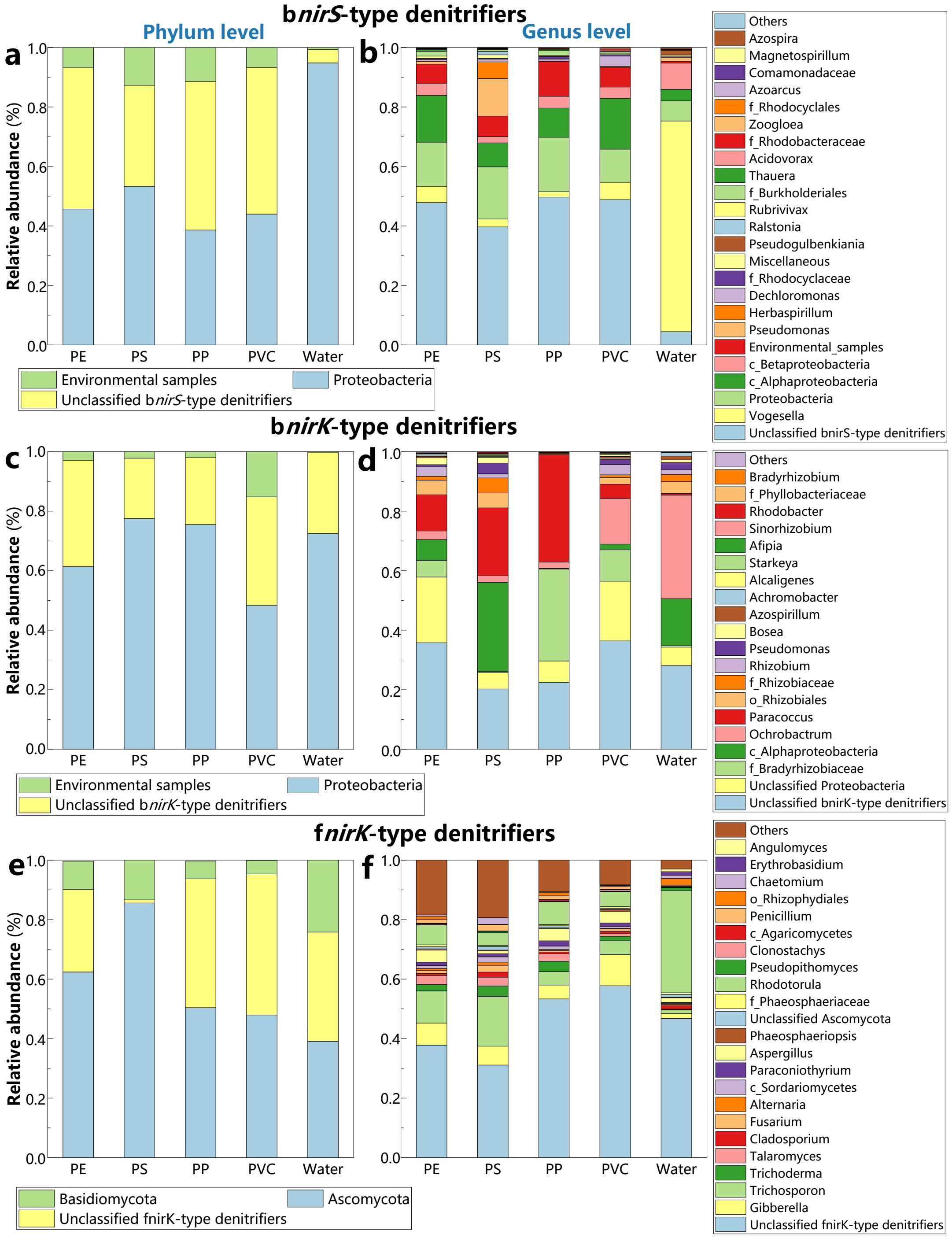

Supplementary Fig. 7. Denitrifier communities in the plastisphere and bulk water. (**a-b).** Phylum and Genus levels of b*nirS*-type denitrifiers. (**c-d).** Phylum and Genus levels of b*nirK*-type denitrifiers. (**e-f).** Phylum and Genus levels of f*nirK*-type denitrifiers.

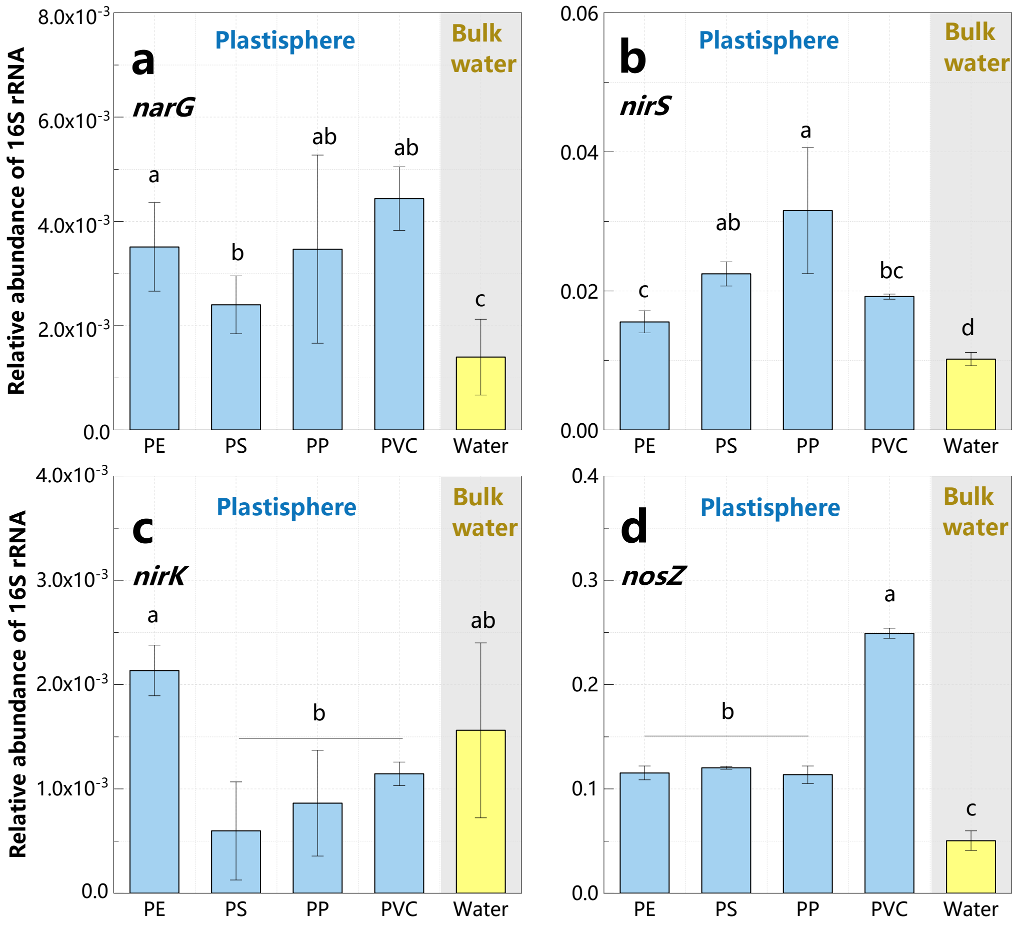

Supplementary Fig. 8. Relative abundances of bacterial denitrifying genes in the plastisphere and bulk water. (**a**). *narG*. (**b**). *nirS*. (**c**). *nirK*. (**d**). *nosZ*. Error bars are the standard error. Different letters (a, b, c, and d) indicate the significant differences ($P<0.05$) between plastisphere and bulk water.

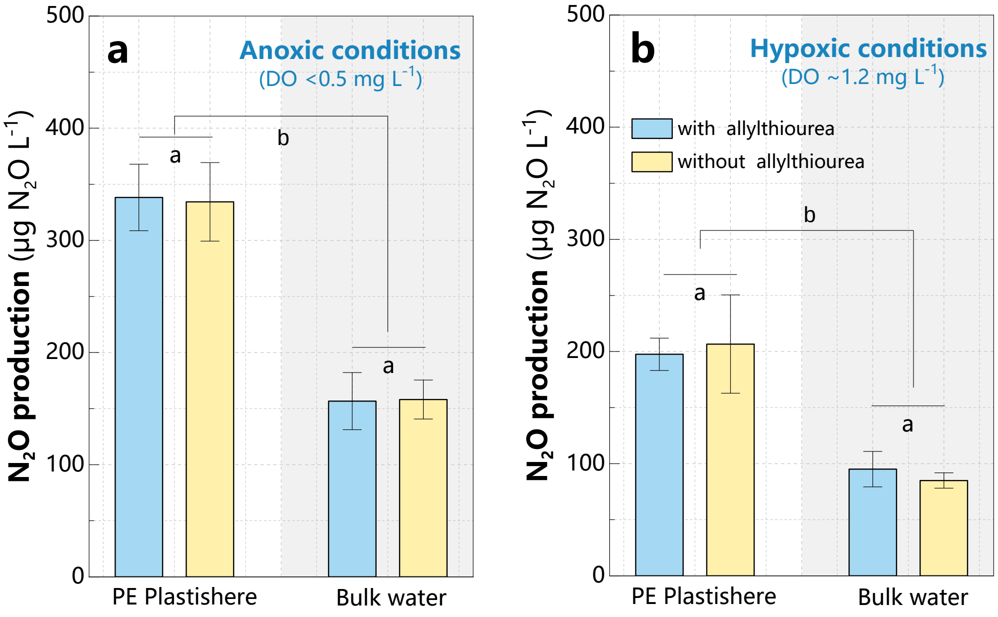

Supplementary Fig. 9. N_2O production in the plastisphere and bulk water groups under anoxic (a) and hypoxic (b) conditions with and without adding allylthiourea. Allylthiourea is a nitrifier inhibitor and can inhibit the activity of ammonia mono-oxygenase (catalyzing ammonia to hydroxylamine) of ammonia-oxidizing archaea (AOA) and bacteria (AOB)[13]. Anoxic conditions were achieved by purging He gas (>99.99%); Hypoxic conditions were achieved by purging He (95%) and O_2 (5%) gases. PE plastisphere was selected as a representation of plastisphere group. Error bars are the standard error. Different letters (a and b) indicate the significant differences ($P<0.05$) between the plastisphere and bulk water groups, and the groups with and without adding allylthiourea.

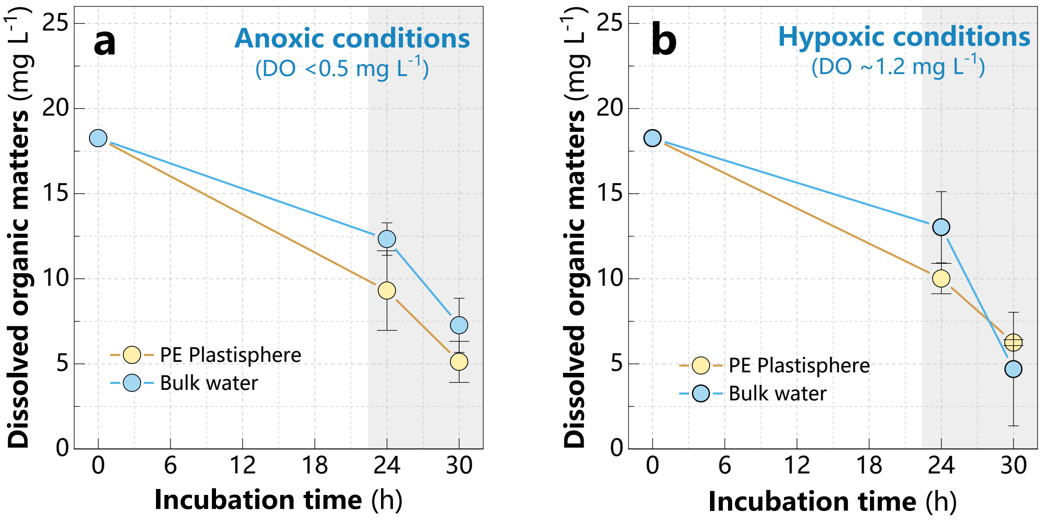

Supplementary Fig. 10. A supplemented 30h experiment showing the changes in the concentrations of dissolved organic matters (DOM) in the plastisphere and bulk water groups under both anoxic (a) and hypoxic (b) conditions. Anoxic conditions were achieved by purging with He gas (>99.99%); Hypoxic conditions were achieved by purging with He (95%) and O_2 (5%) gases. PE plastisphere was selected as a representation of plastisphere group. Concentrations of DOM were measured with a TOC analyzer (TOC-L, SHIMADZU, Japan) after filtering 0.22μm membrane. Error bars are the standard error.

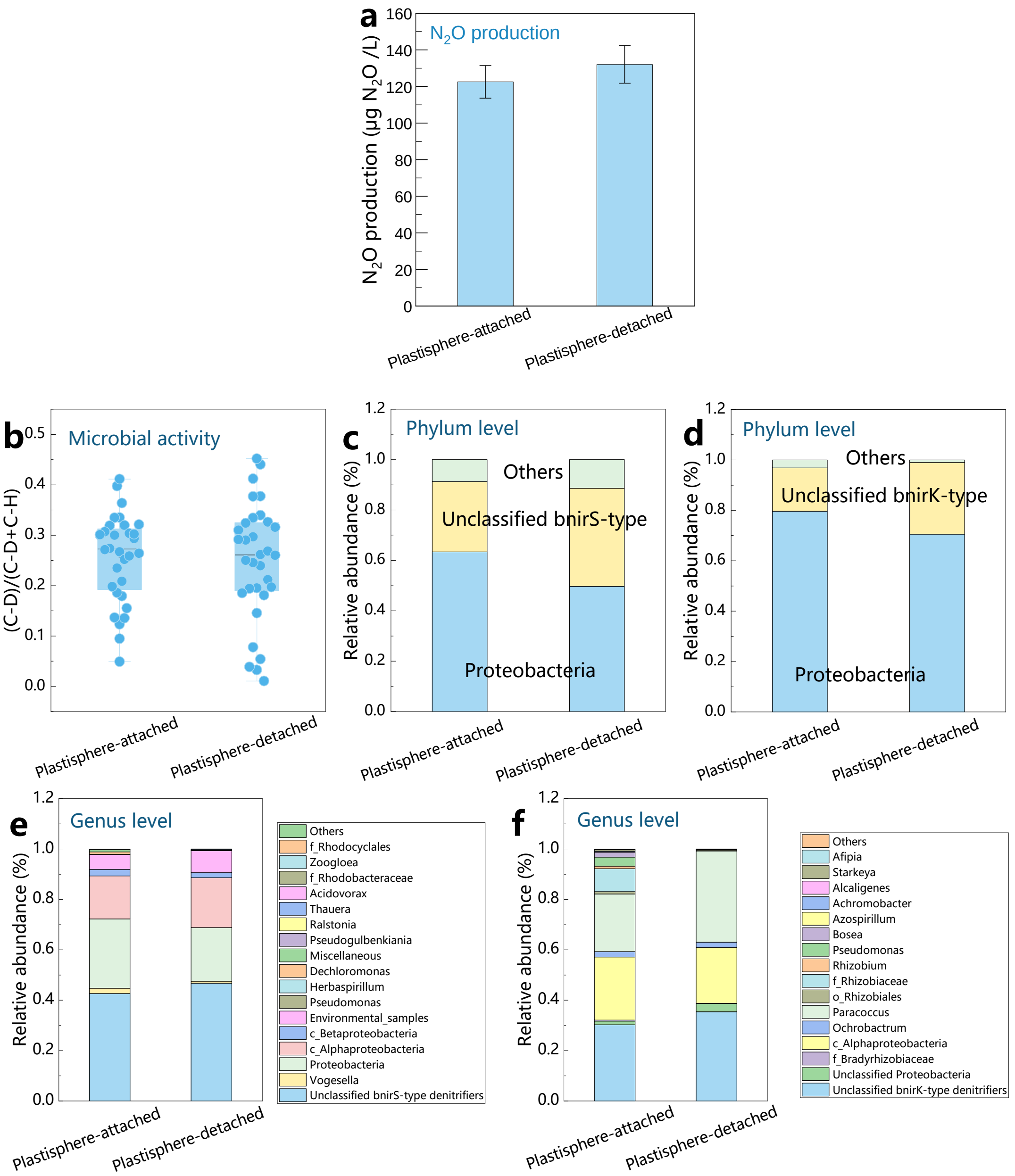

Supplementary Fig. 11. N_2O production, anoxic microbial activities and denitrifier community compositions in attached and detached biofilms after the 24h lab-scale experiment. (**a**). N_2O productions. (**b**). Ratios of (C-D)/(C-D+C-H) calculated by D_2O-single cell Raman technique. (**c-d).** Phylum levels of b*nirS*-type and b*nirK*-type denitrifiers. (**e-f**). Genus levels of b*nirS*-type and b*nirK*-type denitrifiers. This experiment was conducted in 250mL conical flasks, because the plastic debris cannot be placed in the 120mL serum bottles. Error bars are the standard error.

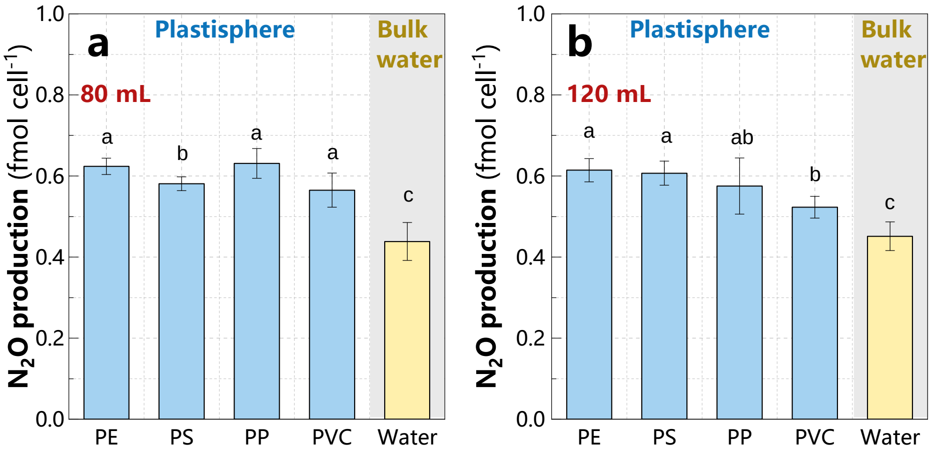

Supplementary Fig. 12. N_2O productions in the plastisphere and bulk water with different water volumes. (**a**). 80 mL. (**b**). 120 mL. Both are normalized with microbial biomasses in the plastisphere and bulk water. Error bars are the standard error. Different letters (a, b, and c) indicate the significant differences ($P<0.05$) among each plastisphere group and bulk water group.

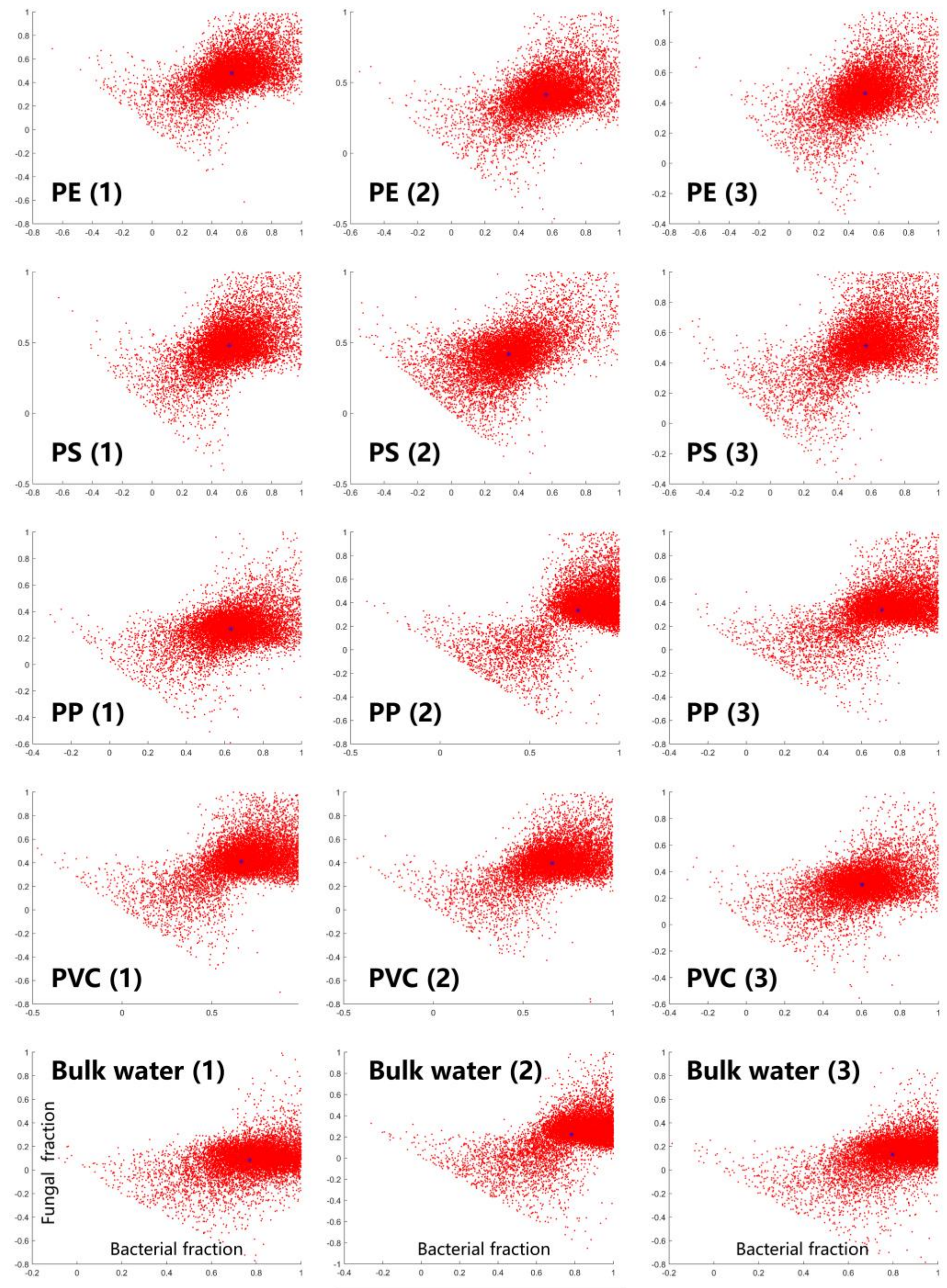

Supplementary Fig. 13. Random sampling (10000 times) during Monte Carlo stimulation. A red dot denotes one sampling, and black dot denotes the mean values of the proportions for BD, FD, and CD processes. X axis is the proportion of f_{BD}, and Y axis is for f_{FD}. f_{CD} equals to 1-f_{BD} and f_{FD}.

Supplementary References

1. Sutka, R. L.; Ostrom, N. E.; Ostrom, P. H.; Breznak, J. A.; Gandhi, H.; Pitt, A. J.; Li, F. Distinguishing nitrous oxide production from nitrification and denitrification on the basis of isotopomer abundances. Appl. Environ. Microb. 2006; 72 (1):638-644.
2. Yu, L.; Harris, E.; Lewicka-Szczebak, D.; Barthel, M.; Blomberg, M. R. A.; Harris, S. J.; Johnson, M. S.; Lehmann, M. F.; Liisberg, J.; Müller, C.; Ostrom, N. E.; Six, J.; Toyoda, S.; Yoshida, N.; Mohn, J. What can we learn from N_2O isotope data? – Analytics, processes and modelling. Rap. Commun. Mass Spectrom. 2020; 34 (20):e8858.
3. Toyoda, S.; Mutobe, H.; Yamagishi, H.; Yoshida, N.; Tanji, Y. Fractionation of N_2O isotopomers during production by denitrifier. Soil Biol. Biochem. 2005; 37 (8):1535-1545.
4. Sutka, R. L.; Adams, G. C.; Ostrom, N. E.; Ostrom, P. H. Isotopologue fractionation during N_2O production by fungal denitrification. Rap. Commun. Mass Spectrom. 2008; 22 (24):3989-3996.
5. Jones, L. C.; Peters, B.; Pacheco, J. S. L.; Casciotti, K. L.; Fendorf, S. Stable isotopes and iron oxide mineral products as markers of chemodenitrification. Environ. Sci. Technol. 2015; 49 (6):3444-3452.
6. Wei, J.; Ibraim, E.; Bruggemann, N.; Vereecken, H.; Mohn, J. First real-time isotopic characterisation of N_2O from chemodenitrification. Geochim. Cosmochim. Ac. 2019; 267:17-32.
7. Wankel, S. D.; Ziebis, W.; Buchwald, C.; Charoenpong, C.; de Beer, D.; Dentinger, J.; Xu, Z.; Zengler, K. Evidence for fungal and chemodenitrification based N_2O flux from nitrogen impacted coastal sediments. Nat. Commun. 2017; 8.
8. Ostrom, N. E.; Pitt, A.; Sutka, R.; Ostrom, P. H.; Grandy, A. S.; Huizinga, K. M.; Robertson, G. P. Isotopologue effects during N_2O reduction in soils and in pure cultures of denitrifiers. J. Geophys. Res. Biogeosci. 2007; 112 (G2):1-12.
9. Ye, C.; Cheng, X. L.; Zhang, K. R.; Du, M.; Zhang, Q. F. Hydrologic pulsing affects

denitrification rates and denitrifier communities in a revegetated riparian ecotone. Soil Biol. Biochem. 2017; 115:137-147.

10. Li, W.; Wang, M. M.; Wang, X. G.; Cheng, X. L.; Guo, J. J.; Bian, X. M.; Cai, L. Fungal communities in sediments of subtropical Chinese seas as estimated by DNA metabarcoding. Sci. Rep. 2016; 6.
11. Lopez-Gutierrez, J. C.; Henry, S.; Hallet, S.; Martin-Laurent, F.; Catroux, G.; Philippot, L. Quantification of a novel group of nitrate-reducing bacteria in the environment by real-time PCR. J. Microbiol. Meth. 2004; 57 (3):399-407.
12. Henry, S.; Bru, D.; Stres, B.; Hallet, S.; Philippot, L. Quantitative detection of the nosZ gene, encoding nitrous oxide reductase, and comparison of the abundances of 16S rRNA, narG, nirK, and nosZ genes in soils. Appl. Environ. Microb. 2006; 72 (8):5181-5189.
13. Ouyang, L.; Thamdrup, B.; Trimmer, M. Coupled nitrification and N_2 gas production as a cryptic process in oxic riverbeds. Nat. Commun. 2021; 12 (1).
