# Supplementary material for: Estuarine plastisphere as an overlooked source of N2O production
Source: Nat Commun. 2022 Jul 6;13:3884. doi: 10.1038/s41467-022-31584-x (PMC9259610; doi:10.1038/s41467-022-31584-x)
Supplement: Supplementary file 3 — Reporting Summary [file 41467_2022_31584_MOESM3_ESM.pdf]

nature portfolio

Corresponding author(s): Yong-guan Zhu

Last updated by author(s): Jun 9, 2022

Reporting Summary

Nature Portfolio wishes to improve the reproducibility of the work that we publish. This form provides structure for consistency and transparency in reporting. For further information on Nature Portfolio policies, see our Editorial Policies and the Editorial Policy Checklist.

Statistics

For all statistical analyses, confirm that the following items are present in the figure legend, table legend, main text, or Methods section.

n/a	Confirmed	
☐	☒	The exact sample size (*n*) for each experimental group/condition, given as a discrete number and unit of measurement
☐	☒	A statement on whether measurements were taken from distinct samples or whether the same sample was measured repeatedly
☐	☒	The statistical test(s) used AND whether they are one- or two-sided<br>*Only common tests should be described solely by name; describe more complex techniques in the Methods section.*
☒	☐	A description of all covariates tested
☒	☐	A description of any assumptions or corrections, such as tests of normality and adjustment for multiple comparisons
☐	☒	A full description of the statistical parameters including central tendency (e.g. means) or other basic estimates (e.g. regression coefficient) AND variation (e.g. standard deviation) or associated estimates of uncertainty (e.g. confidence intervals)
☐	☒	For null hypothesis testing, the test statistic (e.g. F, t, r) with confidence intervals, effect sizes, degrees of freedom and P value noted<br>*Give P values as exact values whenever suitable.*
☐	☒	For Bayesian analysis, information on the choice of priors and Markov chain Monte Carlo settings
☒	☐	For hierarchical and complex designs, identification of the appropriate level for tests and full reporting of outcomes
☒	☐	Estimates of effect sizes (e.g. Cohen's *d*, Pearson's *r*), indicating how they were calculated

Our web collection on statistics for biologists contains articles on many of the points above.

Software and code

Policy information about availability of computer code

Data collection	Data in this study were collected from IRMS, LC-MS/MS, GC-ECD, ICP-MS, IC, and Illumina sequencing platform. Other details see the Materials and methods section.
Data analysis	Most of the analyses were carried out using SPSS (version 22.0) and R (version 3.1.2) softwares; Adobe Illustrator (version CS6) and Origin (version 2019) was used for creating figures; QIIME2 (version 2018.11) was used to bacterial and fungal denitrifier sequences analyses. Additional details are described in the Materials and methods section

For manuscripts utilizing custom algorithms or software that are central to the research but not yet described in published literature, software must be made available to editors and reviewers. We strongly encourage code deposition in a community repository (e.g. GitHub). See the Nature Portfolio guidelines for submitting code & software for further information.

Data

Policy information about availability of data

All manuscripts must include a data availability statement. This statement should provide the following information, where applicable:

- Accession codes, unique identifiers, or web links for publicly available datasets
- A description of any restrictions on data availability
- For clinical datasets or third party data, please ensure that the statement adheres to our policy

The data and the codes supporting the findings of this study are available within the Supplementary Information and Source Data file.

Field-specific reporting

Please select the one below that is the best fit for your research. If you are not sure, read the appropriate sections before making your selection.

☐ Life sciences ☐ Behavioural & social sciences ☒ Ecological, evolutionary & environmental sciences

For a reference copy of the document with all sections, see nature.com/documents/nr-reporting-summary-flat.pdf

Ecological, evolutionary & environmental sciences study design

All studies must disclose on these points even when the disclosure is negative.

Study description	In this study, we chose four types of plastics (PE,PS, PP, PVC) and established in situ and lab-scale incubations in an estuary of Xiamen, China to investigate bacterial denitrification (BD), fungal denitrification (FD), and chemodenitrification (CD) potential in the plastisphere and to differentiate the contributions of these processes to N2O production. We hypothesized that (i) the surface of plastic debris could be a site of plastisphere formation that provides the necessary conditions for denitrification, (ii) plastisphere has higher denitrifying activity than surrounding bulk water, and (iii) FD and CD processes have a great contribution to N2O production relative to BD. we first measured messenger cyclic di-GMP, extracellular polymeric substances (EPS), and intracellular lipid/fatty acid levels to explore the likely mechanism underpinning plastisphere formation. Then the denitrifying activities in plastisphere and bulk water were detected by 15N isotope pairing technique, and the relative contributions of BD, FD, and CD to total N2O production were estimated using N2O isotopocules analysis. Finally, the keystone bacterial and fungal denitrifiers of plastisphere and bulk water were identified. Each group was presented in triplicate.
Research sample	Plastic debris and in situ bulk water were the research samples in this study.
Sampling strategy	For this study, four types of plastisphere and in situ estuarine water were collected (Details see Materials and methods section).
Data collection	S.X., Y.L., T.Y., Y.K., P.J., and Z.Y. executed the incubations, analyzed the results. S.X. and W.Y. analyzed community data via llumina sequencing platform. S.X. and Y.L, measured N speciation via IC and GC-ECD. W.T. and L.R. detected N2O isotopes via IRMS. S.X. and Y.K. conducted single-cell Raman analysis via a LabRAM Aramis. More details see the Materials and methods section.
Timing and spatial scale	All samples and experiments were collected and conducted between March and August 2021.
Data exclusions	No data exclusion
Reproducibility	All measurements were carried out in triplicate or in quintuplicate. All samples were repeatedly collected from each plastisphere and bulk water. The main findings of this study can be reproduced.
Randomization	Samples for each measurements were randomly collected in this study.
Blinding	As both the experiments and the analyses were carried out by the same group of scientists, blinding was not relevant for this study.

Did the study involve field work? ☒ Yes ☐ No

Field work, collection and transport

Field conditions	For this study, in situ incubation was conducted in an estuary of Xiamen, China (118°07'E, 24°61'N~118°09E', 24°59'N) during April and May 2021. This region possesses a subtropical maritime monsoon climate, and has an average temperature of 21°C and receives and 1100 mm of rain. Owing to human activities, pollutants in rainwater runoff and a portion of wastewater have been recently transported to the estuary, leading to reduced water quality and slight eutrophication.
Location	An estuary of Xiamen, China (118°07'E, 24°61'N~118°09E', 24°59'N) .
Access & import/export	The collections of plastic debris and estuarine water in this study did not involve sensitive or prohibited areas.
Disturbance	No disturbance was caused by this study.

Reporting for specific materials, systems and methods

We require information from authors about some types of materials, experimental systems and methods used in many studies. Here, indicate whether each material, system or method listed is relevant to your study. If you are not sure if a list item applies to your research, read the appropriate section before selecting a response.

Materials & experimental systems

n/a	Involved in the study
☒	☐ Antibodies
☒	☐ Eukaryotic cell lines
☒	☐ Palaeontology and archaeology
☒	☐ Animals and other organisms
☒	☐ Human research participants
☒	☐ Clinical data
☒	☐ Dual use research of concern

Methods

n/a	Involved in the study
☒	☐ ChIP-seq
☒	☐ Flow cytometry
☒	☐ MRI-based neuroimaging

March 2021
